# Investigation of possible underlying mechanisms behind water-induced glucose reduction in adults with high copeptin

**DOI:** 10.1038/s41598-021-04224-5

**Published:** 2021-12-29

**Authors:** Sofia Enhörning, Tiphaine Vanhaecke, Alberto Dolci, Erica T. Perrier, Olle Melander

**Affiliations:** 1 Department of Clinical Science, Clinical Research Center, Lund University, Skåne University Hospital, Jan Waldenströms gata 35, 91:12, 214 28 Malmö, Sweden; 2 Department of Internal Medicine, Skåne University Hospital, 205 02 Malmö, Sweden; 3 Danone Research Water Science Team, Danone Research, Route Départementale 128, 91767 Palaiseau, France

**Keywords:** Peptide hormones, Risk factors, Biomarkers, Metabolic disorders, Endocrine system and metabolic diseases

## Abstract

Elevated copeptin, a surrogate marker of vasopressin, is linked to low water intake and increased diabetes risk. Water supplementation in habitual low-drinkers with high copeptin significantly lowers both fasting plasma (fp) copeptin and glucose. This study aims at investigating possible underlying mechanisms. Thirty-one healthy adults with high copeptin (> 10.7 pmol·L^−1^ (men), > 6.1 pmol^−1^ (women)) and 24-h urine volume of < 1.5L and osmolality of > 600 mOsm·kg^−1^ were included. The intervention consisted of addition of 1.5 L water daily for 6 weeks. Fp-adrenocorticotropic hormone (ACTH), fp-cortisol, 24-h urine cortisol, fasting and 2 h (post oral glucose) insulin and glucagon were not significantly affected by the water intervention. However, decreased (Δ baseline-6 weeks) fp-copeptin was significantly associated with Δfp-ACTH (r = 0.76, *p* < 0.001) and Δfp-glucagon (r = 0.39, *p* = 0.03), respectively. When dividing our participants according to baseline copeptin, median fp-ACTH was reduced from 13.0 (interquartile range 9.2–34.5) to 7.7 (5.3–9.9) pmol L^−1^, *p* = 0.007 in the top tertile of copeptin, while no reduction was observed in the other tertiles. The glucose lowering effect from water may partly be attributable to decreased activity in the hypothalamic–pituitary–adrenal axis.

ClinicalTrials.gov: NCT03574688.

## Introduction

Vasopressin (VP) is the key hormone involved in body fluid balance by maintaining constant plasma osmolality. This is achieved by osmoreceptor-stimulated VP release mediating water reabsorption through VP 2 (V2) receptors in the renal collecting ducts in conditions of low water intake^1^. Furthermore, VP is released by changes in blood volume and blood pressure due to stimulation of baroreceptors in the heart and aorta. However, the osmoreceptor-driven VP release is far more sensitive than the baroreceptor-driven release. For example a reduction in plasma volume of 5–10% is shown to have very small effects on VP secretion^1^.

An increasing body of evidence suggests that VP plays a role in glucose homeostasis. Much of the work on VP secretion and its pleiotropic effects has been made possible by the assays that allow analysis of the stable c-terminal fragment of the VP precursor called copeptin^2^. We and others previously established VP as a novel risk marker of metabolic and cardiovascular disease (CVD) development. High copeptin in plasma independently predicts type 2 diabetes^3,4^. Furthermore, high copeptin is associated with the metabolic syndrome, obesity, fatty liver, microalbuminuria and diabetic kidney disease^5–9^, and is shown to be an independent risk factor for CVD^10^. The exact mechanisms that underlie these associations are not known. Neither is it known whether a causal association between the VP system and disease development exists. However, both an experimental study in rats and a human study using a Mendelian randomization approach suggests causality between elevated VP and glucose in plasma^11,12^.

There are many potential ways in which VP could influence glucose and lipid metabolism and cardiovascular risk. Stimulation of VP 1a (V1a) receptors increases gluconeogenesis and glycogenolysis in the liver^13,14^ and platelet aggregation and vasoconstriction in the vessels^15,16^. VP 1b (V1b) receptors mediate adrenocorticotropic hormone (ACTH) release from the anterior pituitary^17^, cortisol secretion from the adrenal gland^18^ and glucagon secretion from the pancreas^19^*.*

Low water intake is one of the important determinants of high VP/copeptin concentrations in otherwise healthy individuals during normal conditions^20,21^, whereas VP secretion is effectively reduced at high water intake. The most prominent water-induced VP reduction is observed in individuals who are habitual low drinkers exhibiting low urine volume, high urine osmolality and high plasma VP^21^. It is thus of interest to investigate the role of increased water intake and decreased VP load on metabolic health. In rats genetically prone to obesity, we demonstrated that water suppressed VP secretion and reversed a dysmetabolic state of insulin resistance and hepatic fat accumulation^12^. In humans, high water intake has been associated with a favourable metabolic profile^22^ and decreased diabetes risk^23,24^. Taken together, these results suggest that the link between high VP and cardiometabolic risk may be modifiable by increased water intake. In the ongoing H2O Metabolism Trial (NCT03422848) we therefore investigate the potential of drinking water to reduce cardiometabolic risk in habitually low-drinking adults with high VP. Our published pilot study showed that 1.5L of increased water intake per day during 6 weeks significantly lowered not only copeptin secretion but also fp glucose^25^.

Given the pleiotropic effects of VP mediated through different receptors in several target organs, this study aims at investigating possible underlying mechanisms behind the glucose reduction observed in the pilot study after 6 weeks of VP-suppressing water treatment and includes a detailed investigation of hormones involved in glucose regulation.

## Methods

Individuals aged 20 to 75 with high plasma copeptin concentrations (> 6.1 pmol L^−1^ in women and > 10.7 pmol L^−1^ in men), corresponding to the top quartile of copeptin in the population-based Malmö Diet and Cancer—Cardiovascular Cohort^3^, were eligible for the current pilot study. They were all previous participants of the Malmö Offspring Study, a population study in the Scania region of southern Sweden. In this cohort, plasma samples were obtained after an overnight fast between the years 2013–2015 and frozen at − 80 °C. Copeptin was measured in these plasma samples. We reached out to 277 individuals with high plasma copeptin with an invitation to participate in the current study. Out of these, 34 individuals were included. In addition to high copeptin and age range as specified above, inclusion criteria were laboratory findings that, together with high copeptin, indicated low water intake (24-h urine osmolality ≥ 600 mOsm · kg · L^−1^ water and 24-h urine volume ≤ 1.5 L). Exclusion criteria were as follows: pregnancy or breastfeeding; plasma sodium < 135 mM; use of diuretics, lithium, or selective serotonin reuptake inhibitors; chronic kidney disease (estimated glomerular filtration rate (eGFR) < 30 mL/min per 1.73 m^2^); heart failure; type 1 diabetes or type 2 diabetes treated with insulin; inflammatory bowel disease; and vulnerable persons (those with a legal guardian or loss of personal liberty). Thus, individuals with increased risk for hyponatremia (*i.e.,* use of diuretics, lithium, or selective serotonin reuptake inhibitors; heart failure) were not eligible to participate for safety reasons. A detailed description of the recruitment and inclusion process of the pilot study can be found elsewhere^25^. The pilot study was designed to investigate the copeptin lowering effect from moderate water intake in low-drinkers, as well as to test study logistics, safety, compliance, and drop-out rate before the start of the long-term H2O Metabolism Trial. To reach these objectives, a sample size of ~ 30 individuals was judged to be satisfactory.

All participants provided written informed consent before their inclusion in the study. The study was approved by the ethics committee of Lund University and performed in accordance with the ethical standards laid down in the 1964 Declaration of Helsinki and its later amendments.

Study visits were performed at baseline and at the end of the 6-week intervention. Participants arrived at the clinic between 7:45 am and 9:15 am after an overnight fast. The study protocol was previously described in detail^25^. At the study visits, measurements of copeptin, glucose, glucagon, insulin, ACTH and cortisol were performed sitting in an upright position. Furthermore, the insulin and glucagon response to an oral glucose tolerance test (OGTT) was assessed after 120 min. At baseline and after 6 weeks, fluid and dietary intakes were assessed using a previously validated web-based 4-day record tool^25,26^.

Participants collected 24-h urine samples the day before each study visit. Upon awakening, participants voided and discarded this first morning urine sample. All subsequent urine produced throughout the day and overnight was collected in a single container. The next morning, upon awakening, participants produced a final first morning void, which completed the 24-h collection. Participants delivered the complete, fresh 24-h urine samples to the clinic during their study visit that day. The 24-h urine collections followed procedures developed at the Department of Endocrinology, Skåne University Hospital Malmö, Sweden, and consisted of a comprehensible written instruction aimed at ensuring accurate and complete collection of urine.

### Laboratory measurements

Fasting plasma (fp) copeptin concentration was measured by using a KRYPTOR Compact Plus device and commercially available chemiluminescence sandwich immunoassay copeptin ProAVP kit with coated tubes from samples stored at − 80 °C (BRAHMS Copeptin proAVP KRYPTOR; ThermoFisher Scientific). Plasma glucose, ACTH and cortisol, serum insulin and urine cortisol measurements were performed at the certified University Hospital’s central clinical laboratory. Plasma glucagon concentration was measured at the Biomedical centre, Lund University, by using a commercially available immunoassay from samples stored at − 80 °C (Mercodia sandwich ELISA).

### Statistics

Significance of differences between baseline and after intervention was tested using Wilcoxon paired rank test. Correlation coefficients between delta (Δ) values (difference in change between baseline and 6-week values) were determined by using Pearson correlation. Values of copeptin, ACTH and urine cortisol were ln-transformed before the Δ values were calculated to avoid non-normally distributed Δ values. Subjects were a posteriori divided into tertiles of baseline fp copeptin. Finally, significant differences in change of fp ACTH and urine cortisol were tested in subgroups defined based on baseline copeptin using one sample *t *test. SPSS statistical software version 26 (SPSS Inc., Chicago, Ill., USA) was used for all analyses. A 2-sided *p* value of < 0.05 was considered statistically significant.

## Results

Out of the 24% of the individuals participating in the Malmö Offspring Study with high copeptin concentrations (> 6.1 pmol·L^−1^ in women and > 10.7 pmol·L^−1^ in men), 277 were contacted, and 34 individuals were eligible and included in the study (82 were not reached, 93 were not interested and 38 were excluded; out of 64 qualified individuals, 30 could not be included due to either low urine osmolality or high urine volume). Three individuals dropped out during the intervention. In two cases the drop out was due to infections without any suspicion of link to the ongoing water intervention. In one case the drop out was for personal reasons. The mean age among the 31 individuals that completed the 6-week water intervention was 43 (min 22, max 66) years and 61% were men. As previously described, the anticipated extra 1.5 L of water per day during 6 weeks resulted in an achieved extra water intake and increased urine volume of around 1.2 L^25^.

### The impact of water on glucoregulatory hormones

The 6-week water intervention did not result in any significant change in either fp ACTH, fp cortisol, fasting and 2 h-OGTT insulin or glucagon or urine cortisol concentrations (Table 1, Supplementary Fig. 1) in parallel with the previously described decrease in fp copeptin and fp glucose. However, the water-induced change in fp copeptin between baseline and 6 weeks significantly correlated with a water-induced change in fp ACTH and fp glucagon, respectively (Table 2). Change in fp copeptin (baseline—6 weeks) did not correlate with a change in any of the other measured glucoregulatory hormones (Table 2).

**Table 1:** Measures of water intake, ACTH, cortisol, insulin and glucagon at baseline and after water intervention (n = 31).

	Baseline	After 6 weeks	*P* value
p-copeptin (pmol/L)	12.9 (7.4–21.9)	7.8 (4.6–11.3)	< 0.001
u-osmolality (mOsm/kg H_2_O)	879 (705–996)	384 (319–502)	< 0.001
u-volume (L/24 h)	1.06 (0.90–1.20)	2.27 (1.52–2.67)	< 0.001
Drinking water (L/day)	0.43 (0.27–0.58)	1.35 (1.00–1.71)	< 0.001
p-glucose (mmol/L)	5.9 (5.6–6.3)	5.6 (5.4–6.0)	0.03
p-ACTH (pmol/L)	4.6 (3.4–13.0)	5.4 (3.8–8.9)	0.36
p-cortisol (nmol/L)	309 (265–401)	342 (252–412)	0.39
u-cortisol (nmol/24 h)	70.9 (48.7–85.8)^a^	77.2 (45.9–119.9)^b^	0.08
s-insulin fasting (mIE/L)	10.0 (8.0–14.0)	11.0 (6.0–15.0)	0.27
s-insulin 120 min post OGTT (mIE/L)	50.0 (29.0–96.0)	49.0 (27.0–71.0)	0.52
p-glucagon fasting (pmol/L)	10.17 (8.15–12.36)	10.22 (6.67–14.36)	0.80
p-glucagon 120 min post OGTT (pmol/L)	3.86 (2.44–6.58)	4.38 (2.46–7.02)	0.48

Data given as median [25th;75th percentiles] and *P* value calculated from Wilcoxon paired rank test.

^a^N = 29.

^b^ N = 30.

**Table 2:** Correlations between Δ change of copeptin (baseline—6 weeks) and Δ change of metabolic variables.

	Correlation (Pearson’s r)	*P* value
Δ fp-glucose	0.30	0.11
Δ p-ACTH	0.76	< 0.001
Δ p-cortisol	0.24	0.19
Δ u-cortisol (pmol/24 h)^a^	0.31	0.11
Δ p-glucagon, fasting	0.39	0.03
Δ p-glucagon, 120 min post OGTT	0.16	0.39
Δ s-insulin, fasting	-0.04	0.82
Δ s-insulin, 120 min post OGTT	0.16	0.39

ΔCopeptin, ΔACTH and Δu-Cortisol were skewed to the right and therefore values at baseline and after water intervention were ln-transformed before the Δ value was calculated.

^a^N = 28.

### Stratification in tertiles of baseline copeptin

**Figure 1:**
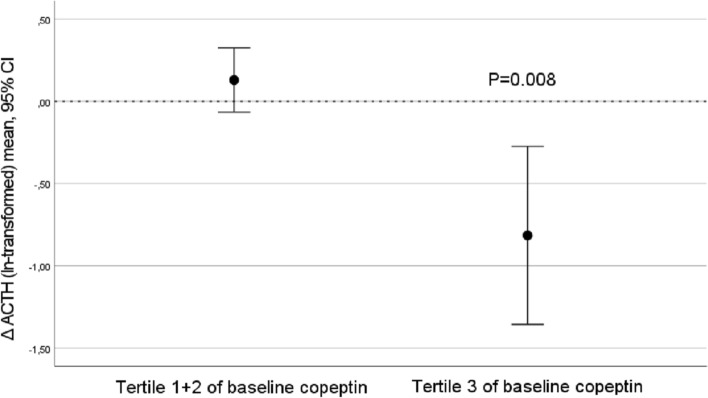
ACTH reduction after water intervention was evident among individuals (n = 10) belonging to the top tertile of pilot baseline copeptin concentration.

As we previously found that participants in the top tertile of baseline copeptin had the best water-induced copeptin and glucose reduction, we stratified our population in tertiles of baseline copeptin_,_ and found that in the top tertile, fp ACTH was reduced from a median of 13.0 (interquartile range 9.2–34.5) to 7.7 (5.3–9.9) pmol·L^−1^, *p* = 0.007, while no fp ACTH reduction was observed in the other copeptin tertiles (Fig. 1, Supplementary Table 1, Supplementary Fig. 1). Furthermore, a water-induced increase in urine cortisol was observed among individuals belonging to tertile 1 and 2 of baseline copeptin, while no such increase was observed in the top tertile of baseline copeptin (Supplementary Fig. 2). None of the other investigated glucoregulatory hormones were significantly affected by the intervention in any tertile of baseline copeptin (Supplementary Table 1).

### Correlation between glucose reduction and other glucoregulatory hormones

Finally, when investigating correlations between water-induced change in fp glucose (baseline—6 weeks) and change in other glucoregulatory hormones, we found that the water-induced change in fp glucose was significantly associated with water-induced change in fp cortisol, while there were borderline significant correlations between change in fp glucose and change in fp ACTH and 120 min insulin respectively (Table 3). Change in fp glucose did not correlate significantly with change in the other investigated glucoregulatory hormones (Table 3).

**Table 3:** Correlations between Δ change after water treatment (baseline—6 weeks) of glucose and Δ change of other metabolic variables.

	Correlation (Pearson’s r)	*P* value
Δ p-ACTH	0.34	0.06
Δ p-cortisol	0.37	0.04
Δ u-cortisol (pmol/24 h)^a^	0.21	0.28
Δ p-glucagon, fasting	0.08	0.66
Δ p-glucagon, 120 min post OGTT	0.12	0.51
Δ s-insulin, fasting	0.21	0.25
Δ s-insulin, 120 min post OGTT	0.34	0.06

ΔCopeptin, ΔACTH and Δu-Cortisol were skewed to the right and therefore values at baseline and after water intervention were ln-transformed before the Δ value was calculated.

^a^N = 28.

## Discussion

The main finding of this study was that the copeptin reduction (Δ fp-copeptin) observed after the addition of 1.5L of water per day during the 6-week intervention significantly correlated with Δ fp-ACTH, pointing at a possible underlying mechanism behind the glucose reduction previously observed in the same study upon water treatment. Fp-ACTH reduction was observed only in the one-third of the participants with the highest baseline copeptin concentration. As described in our previous publication^25^, there were no changes in weight or dietary intake (except from increased water intake) between baseline and after 6 weeks that could possibly explain our results. Additionally, there were no differences between tertiles of baseline copeptin regarding either age, body mass index, sodium levels, plasma osmolality or C reactive protein that could have influenced our findings. Interestingly, this top tertile of baseline copeptin is the same subset of the participants that also showed the best glucose lowering effect from water treatment^25^, suggesting that water supplementation may be particularly important in individuals with high plasma copeptin and thus increased cardiometabolic risk.

VP is known to mediate ACTH secretion upon stressful stimuli through V1bR in the anterior pituitary gland, which in turn elevates glucocorticoids in plasma^17^. This VP-induced ACTH release has been reported to be resistant to glucocorticoid feedback in contrast to the corticotropin-releasing hormone-induced ACTH release^27^. Furthermore, copeptin has previously been established as a risk marker of several Cushing-related conditions^3,5,10^. Therefore it can be speculated that excessive VP release, induced by for example low water intake, overstimulates the hypothalamic–pituitary–adrenal (HPA) axis and elevates glucocorticoid levels, leading to development of a mild Cushing-like phenotype with overweight, insulin resistance, diabetes and increased cardiovascular risk in the long term^28^.

The water-induced copeptin reduction (Δ fp-copeptin) between baseline and 6 weeks significantly correlated not only with Δ fp-ACTH but also with Δ fp-glucagon. However, as compared with the ACTH reduction, no glucagon lowering effect was observed among the individuals with the largest copeptin and glucose lowering effect, i.e., those belonging to the top tertile of baseline copeptin concentration. We therefore argue that it is more likely that the water-induced glucose reduction in the pilot study is associated with an attenuated HPA-axis response rather than a reduction of the diabetogenic hormone glucagon. Nonetheless, the results showing a correlation between Δ fp-copeptin and Δ fp-glucagon are of interest, especially since VP through the V1bR is known to mediate glucagon secretion in man^19^, and since we previously found a reduction of glucagon in low-drinking individuals after 1 week of high water intake^21^. Thus, one cannot rule out that the correlation between Δ fp-copeptin and Δ fp-glucagon observed in this study indicates a beneficial effect from water on glucose homeostasis linked to altered glucagon secretion.

Furthermore, we investigated if the previously observed water-induced glucose reduction correlated with a reduction in any of the investigated glucoregulatory hormones. Interestingly, we found that the reduction in fp glucose between baseline and 6 weeks significantly correlated with fp cortisol reduction, while the correlation with fp ACTH reduction did not hold statistical significance. As ACTH is the main regulator of cortisol secretion, we believe that these findings are in line with the hypothesis that low water intake overstimulates the HPA axis and hereby increases fp glucose, and that this effect can be attenuated by increased water intake.

There was no observed change in urine cortisol excretion at baseline compared to after 6 weeks of increased water intake. In the literature, a rise in urine cortisol when water intake and diuresis increases is previously well described^29,30^. The rise in urine cortisol is not thought to be linked to increased cortisol secretion when water intake increases, but rather to decreased reuptake or metabolism of cortisol in the kidney tubules^29^. Due to this previously established water-induced increase in urine cortisol, we did not expect to find any water-induced reduction in urine cortisol in our study. Instead, and in line with previous literature, a slight increase in urine cortisol was observed, which however was non-significant. In the current and previous water interventions, we have noted that when an additional amount of water is prescribed, the actual increase achieved by participants will be slightly less than the targeted added volume^21,25^. The achieved difference in 24-h urine volume between the baseline and end of intervention in the current study was around 1.2L. Thus, we conclude that an achieved extra water intake of around 1.2L per day is not sufficient to significantly increase urine cortisol concentrations in individuals with habitually low water intake. Because the concentration of free cortisol in urine is dependent on the concentration of p-cortisol, and thus p-ACTH secretion^29^, one may speculate that the overall lack of significant increase in urine cortisol may after all be associated with a water-induced attenuation of p-ACTH and p-cortisol secretion. In support of this theory, we observed a significant increase in urine cortisol in the participants belonging to the bottom two tertiles of baseline copeptin (i.e., individuals without any significant ACTH reduction after water treatment), while no increase in urine cortisol was observed in the top tertile of baseline copeptin (i.e., representing individuals with a significant water-induced decrease in ACTH) (Supplementary Fig. 2).

The renin–angiotensin–aldosterone system is also involved in the regulation of water homeostasis, and extra water intake may have resulted in a suppression of angiotensin II. Moreover, angiotensin II is previously shown to enhance the secretion of ACTH^31^. Unfortunately, no markers of the renin–angiotensin–aldosterone system were measured in this study, which we acknowledge as a limitation. Thus, we cannot rule out the possibility that the observed ACTH reduction among individuals belonging to the top tertile of baseline copeptin concentration was associated with a water induced angiotensin II reduction. As previously reported from this pilot study, increased water intake resulted in a slight but significant fp-sodium reduction [mean (SD) in mmol/L: 141.2 (1.65) at baseline; 140.1 (1.41) at 6 weeks, *p* = 0.001], whereas neither systolic or diastolic blood pressure nor fp-osmolality differed between baseline and the 6 week measurement^25^. It can be speculated that the sodium reduction was a result from a water-induced reduction of angiotensin II.

As this pilot study was not designed or powered to study any water-induced reduction in glucometabolic parameters, but designed to investigate safety and logistics before the start of a long-term water intervention trial, we did not include any control group (i.e. without active water treatment) or comparison group (i.e. low baseline copeptin), which we acknowledge as a limitation.

We conclude that water supplementation in habitually low-drinking individuals with high copeptin concentration lowers both copeptin and glucose concentration, as well as ACTH concentration in individuals belonging to the highest tertile of baseline copeptin. These results point at an attenuated HPA-axis response as a possible explanation behind the positive effect from increased water intake on glucose metabolism.

## Supplementary Information

Supplementary Information.

## Data Availability

The dataset generated and analysed during the current study is available from the corresponding author on reasonable request.
