# Supplementary material for: Investigation of possible underlying mechanisms behind water-induced glucose reduction in adults with high copeptin
Source: Sci Rep. 2021 Dec 29;11:24481. doi: 10.1038/s41598-021-04224-5 (PMC8716535; doi:10.1038/s41598-021-04224-5)

Investigation of possible underlying mechanisms behind water-induced glucose reduction in adults with high copeptin

Sofia Enhörning, Tiphaine Vanhaecke, Alberto Dolci, Erica T. Perrier, Olle Melander

Supplementary Table 1. Copeptin, glucose, ACTH, cortisol, insulin and glucagon at baseline and after water intervention in different tertiles[1] of baseline copeptin.

		Baseline	After 6 weeks	P-value
**Tertile 1** N=10	**p-copeptin (pmol/L)**	6.5 (4.4-9.1)	4.6 (3.8-7.1)	0.09
	**p-glucose (mmol/L)**	5.90 (5.60-6.40)	6.0 (5.40-6.40)	0.046
	**p-ACTH (pmol/L)**	3.8 (3.3-8.7)	4.8 (3.8-8.5)	0.10
	**p-cortisol (nmol/L)**	344 (287-403)	353 (274-433)	0.17
	**u-cortisol (nmol/24h) [2]**	70.9 (49.4-81.3)	75.6 (45.9-122.1)	0.31
	**s-insulin fasting (mIE/L)**	8.5 (6.8-11.0)	7.0 (5.8-16.8)	0.53
	**s-insulin 120 min post OGTT (mIE/L)**	30.0 (24.8-59.5)	37.5 (7.8-71.8)	0.65
	**p-glucagon fasting (pmol/L)**	9.1 (7.4-11.3)	8.8 (5.1-15.5)	0.51
	**p-glucagon 120 min post OGTT (pmol/L)**	3.1 (2.4-3.9)	3.7 (2.3-5.7)	0.26
**Tertile 2** N=11	**p-copeptin (pmol/L)**	12.9 (7.5-15.2)	8.6 (5.1-12.5)	0.09
	**p-glucose (mmol/L)**	5.90 (5.60-6.40)	6.0 (5.40-6.40)	0.91
	**p-ACTH (pmol/L)**	4.1 (3.1-4.6)	4.5 (3.5-7.7)	0.21
	**p-cortisol (nmol/L)**	265 (203-309)	312 (239-373)	0.16
	**u-cortisol (nmol/24h) [2]**	65.6 (41.5-95.6)	87.0 (55.3-122-8)	0.03
	**s-insulin fasting (mIE/L)**	9.0 (7.0-20.0)	8.0 (6.0-15.0)	0.11
	**s-insulin 120 min post OGTT (mIE/L)**	56.0 (46.0-74.0)	39.0 (28.0-80.0)	0.24
	**p-glucagon fasting (pmol/L)**	11.1 (8.1-12.9)	8.7 (7.5-16.7)	0.79
	**p-glucagon 120 min post OGTT (pmol/L)**	4.6 (2.4-6.8)	4.2 (2.3-7.0)	0.42
**Tertile 3** N=10	**p-copeptin (pmol/L)**	35.2 (21.0-66.5)	9.4 (7.0-12.7)	0.005
	**p-glucose (mmol/L)**	5.85 (5.60-6.50)	5.55 (5.30-5.90)	0.03
	**p-ACTH (pmol/L)**	13.0 (9.2-34.5)	7.6 (5.3-9.9)	0.007
	**p-cortisol (nmol/L)**	397 (302-529)	354 (242-524)	0.45
	**u-cortisol (nmol/24h) [2]**	72.7 (52.2-89.0)	57.7 (21.9-116.5)	0.86
	**s-insulin fasting (mIE/L)**	12.0 (10.0-27.0)	13.0 (11.8-17.3)	0.62
	**s-insulin 120 min post OGTT (mIE/L)**	67.5 (23.0-112.8)	56.5 (35.3-85.8)	0.72
	**p-glucagon fasting (pmol/L)**	10.8 (8.2-16.9)	10.8 (8.5-14.7)	0.45
	**p-glucagon 120 min post OGTT (pmol/L)**	5.5 (2.5-14.4)	6.5 (2.4-8.1)	0.33

Data given as median [25th;75th percentiles]. [1] Pooled sex-specific tertiles. [2] N=28 (n tertile 1=9, n tertile 2=10, n tertile 3=9)

Supplementary Figure 1.

Graphs showing individual changes of metabolic parameters between baseline and after 6 weeks of water treatment. Green lines represent individuals belonging to tertile 1, yellow lines represent individuals belonging to tertile 2, and red lines represent individuals belonging to tertile 3 of baseline copeptin.

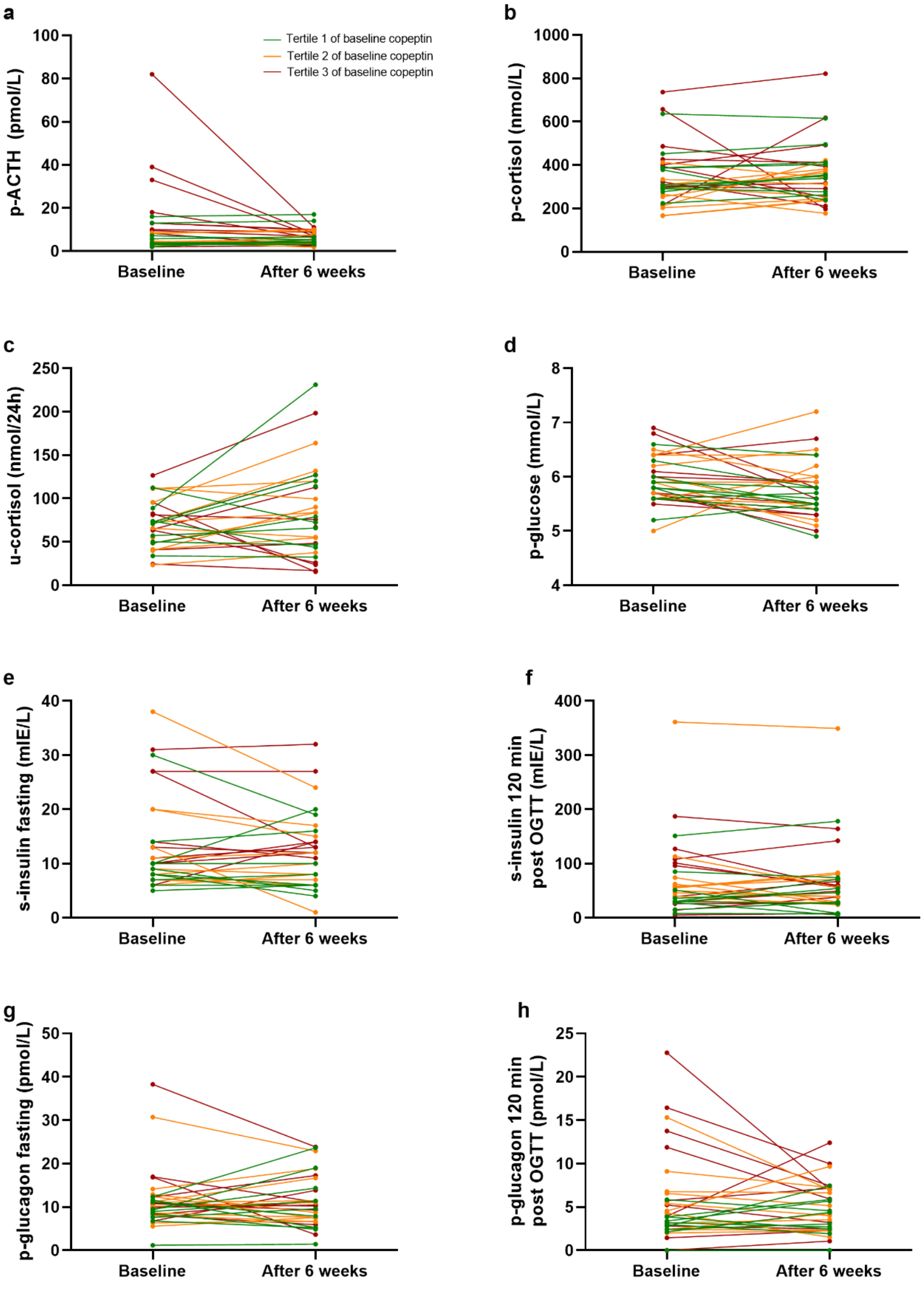

Supplementary Figure 2.
Individuals belonging to the bottom tertiles (n=19) of baseline copeptin had increased Δ urine cortisol while individuals belonging to the top tertile (n=9) of baseline copeptin did not.

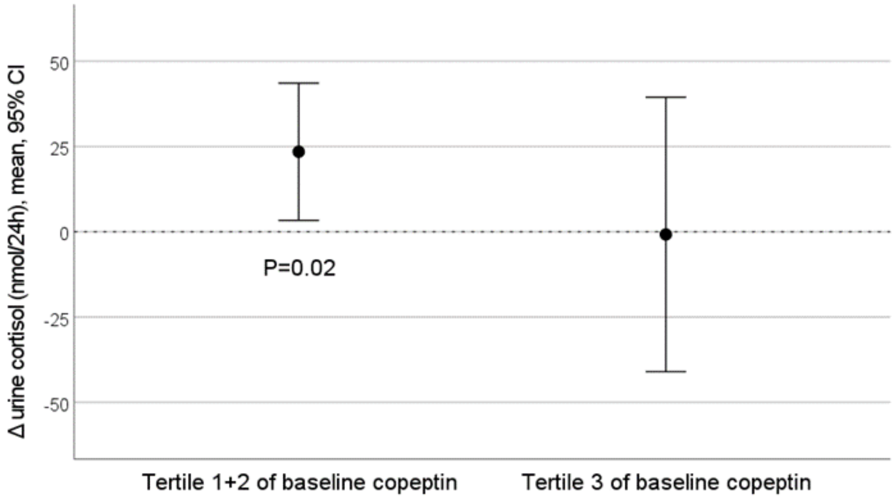

Supplementary Figure 3a.
Scatterplot showing the relationship between Δ fp-copeptin (baseline - 6 weeks) and Δ fp-ACTH (baseline - 6 weeks).

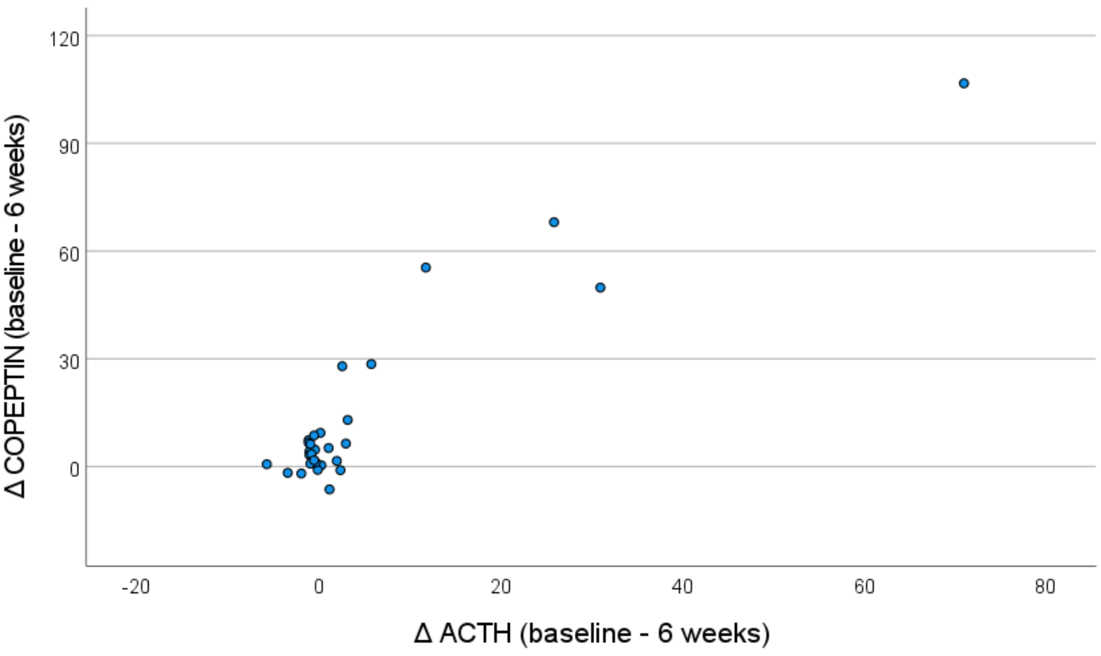

Supplementary Figure 3b.
Scatterplot showing the relationship between Δ fp-copeptin (baseline - 6 weeks) and Δ fp-glucagon (baseline - 6 weeks).

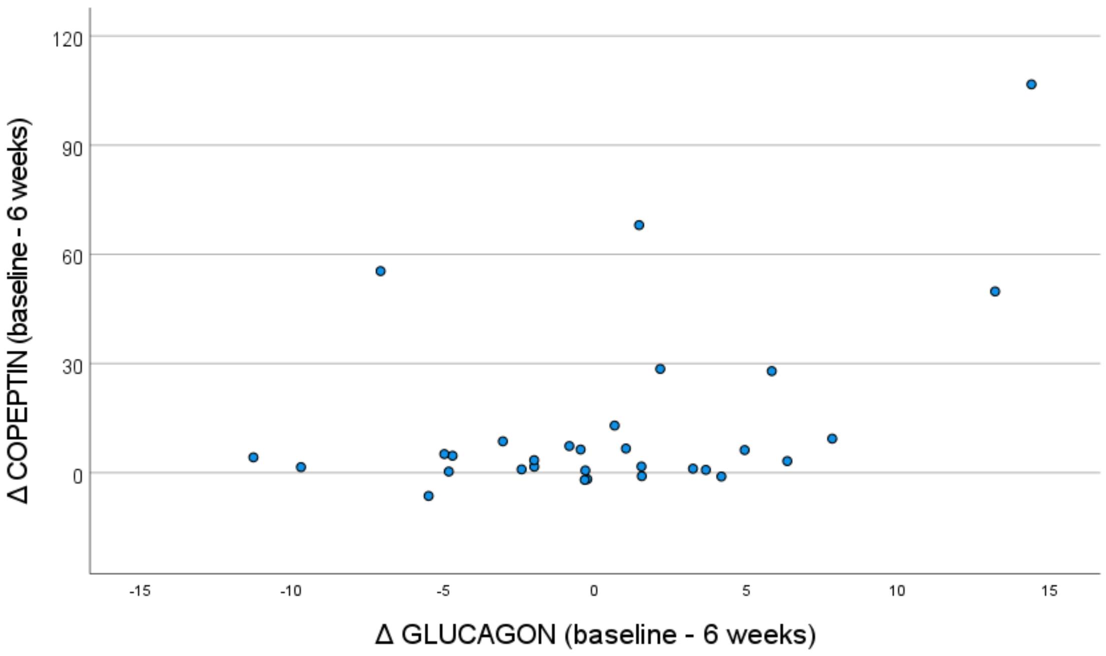

Supplement: Supplementary file 1 — Supplementary Information. [file 41598_2021_4224_MOESM1_ESM.pdf]
